# Clock Genes and Altered Sleep–Wake Rhythms: Their Role in the Development of Psychiatric Disorders

**DOI:** 10.3390/ijms18050938

**Published:** 2017-04-29

**Authors:** Annaëlle Charrier, Bertrand Olliac, Pierre Roubertoux, Sylvie Tordjman

**Affiliations:** 1 Pôle Hospitalo-Universitaire de Psychiatrie de l’Enfant et de l’Adolescent (PHUPEA), Université de Rennes 1, Centre Hospitalier Guillaume-Régnier, 154 Rue de Châtillon, Rennes 35000, France; s.tordjman@yahoo.fr; 2 Pôle Universitaire de Psychiatrie de l’Enfant et de l’Adolescent, Centre Hospitalier Esquirol, Limoges 87025, France; Bertrand.Olliac@chu-limoges.fr; 3 INSERM, U1094, Tropical Neuroepidemiology, Limoges 87000, France; 4 Aix Marseille Université, INSERM, GMGF UMR_S 910, Marseille 13385, France; pierre.roubertoux@univ-amu.fr; 5 Laboratoire Psychologie de la Perception (LPP), Université Paris Descartes, CNRS UMR 8158, Paris 75270, France

**Keywords:** clock genes, circadian rhythm, circadian clocks network, synchronization of oscillators, sleep-wake rhythm, psychiatric disorders, schizophrenia, autism spectrum disorder, mood disorders, attention deficit hyperactivity disorder

## Abstract

In mammals, the circadian clocks network (central and peripheral oscillators) controls circadian rhythms and orchestrates the expression of a range of downstream genes, allowing the organism to anticipate and adapt to environmental changes. Beyond their role in circadian rhythms, several studies have highlighted that circadian clock genes may have a more widespread physiological effect on cognition, mood, and reward-related behaviors. Furthermore, single nucleotide polymorphisms in core circadian clock genes have been associated with psychiatric disorders (such as autism spectrum disorder, schizophrenia, anxiety disorders, major depressive disorder, bipolar disorder, and attention deficit hyperactivity disorder). However, the underlying mechanisms of these associations remain to be ascertained and the cause–effect relationships are not clearly established. The objective of this article is to clarify the role of clock genes and altered sleep–wake rhythms in the development of psychiatric disorders (sleep problems are often observed at early onset of psychiatric disorders). First, the molecular mechanisms of circadian rhythms are described. Then, the relationships between disrupted circadian rhythms, including sleep–wake rhythms, and psychiatric disorders are discussed. Further research may open interesting perspectives with promising avenues for early detection and therapeutic intervention in psychiatric disorders.

## 1. Introduction: Circadian Rhythms and Their Molecular Mechanisms

The function of the body is subject to different biological rhythms, the circadian one being keyed to a cycle of a 24-h day corresponding approximately to the 24-h light/dark cycle of the Earth’s rotation. In fact, the range of the internal clock period for healthy adults is 24 h and 11 ± 16 min but it is set back to a 24-h cycle each day by exposure to morning light and to external clocks [1]. This rhythm regulates most of our biological and behavioral functions. Its dysregulation leads to sleep disorders and major physiological disturbances. In psychiatric disorders, sleep problems (especially reduced total sleep with insomnia, longer sleep latency, nocturnal and early morning awakenings) are highly prevalent symptoms. Insomnia is the most frequent sleep problem reported in psychiatric disorders [2]. In autism, a prevalence of insomnia from 50% to 80% has been reported [3,4,5,6] compared to 9–50% in age-matched typically developing children [7,8,9,10,11]. However, the interpretation of these results has to take into consideration the high prevalence of insomnia (30%) in typically developing individuals [12,13,14]. It is noteworthy that sleep disorders are not specific to autism spectrum disorder (ASD) given that they are observed in ASD individuals with intellectual disability (ID), ID individuals without ASD, and individuals with brain injury as well as ID, suggesting a possible overlap between ASD and ID. Concerning schizophrenia, a genome-wide association study (GWAS) shows genetic correlations between sleep disorders and schizophrenia and between the late evening chronotype and risk of schizophrenia [15]. No rigorous study of prevalence of sleep disorders was conducted in bipolar spectrum disorder and depression-related disorders but altered patterns of clock genes were reported in these disorders [16,17]. Conversely, high rates of sleep problems and mental disorders have been reported in shift workers, including insomnia, fatigue, anxiety and depression [18,19,20,21,22,23,24]. It is noteworthy that altered patterns of clock genes were also reported in shift workers [25,26,27].

The objective of this article is an attempt to clarify the relationships between psychiatric disorders, altered expression of clock genes and endogenous circadian rhythm disturbances (especially altered sleep–wake rhythms). Is the disruption of circadian rhythms, and therefore of sleep–wake rhythms, a prerequisite for the genesis of psychiatric disorders? What roles do the clock genes play in the disruption of endogenous circadian rhythms? After describing the molecular mechanisms of circadian rhythms, their relationships with psychiatric disorders are discussed, focusing in particular on bipolar disorder, anxiety, depression, attention deficit disorder, schizophrenia and autism spectrum disorder (a rich literature review on this topic was available for these psychiatric disorders, which was not the case for other disorders such as addictions or dementia).

### 1.1. Daily and Circadian Rhythms

There are different categories of physiological rhythms with different time scales, such as ultradian, tidal, circadian, lunar, and seasonal rhythms. A daily rhythm is a regular and predictable phenomenon which is defined as a series of significant physiological changes over 24 h. The biological rhythm consists of two components: the first is exogenous and modulated by environmental factors such as light–dark alternations, sleep–wake, hot–cold, and seasonal change [28,29,30,31]; the second is endogenous and linked to genetic factors (studies in identical twins showed that they had identical biological rhythms [32]). Homologous genes involved in the animal’s activity–rest cycle have been described in humans. It has been observed that the individual tendency to get up and go to bed more or less early was associated with a polymorphism of the *circadian locomotor output cycles kaput* (*Clock*) gene. Socio-ecological exogenous factors modulate rhythms and are called synchronizers. Endogenous factors, genetic by nature, underpin the internal biological clock which is responsible for an internal time synchronization coordinating the circadian variations of biochemical, physiological and behavioral parameters.

Circadian rhythms are the best known biological rhythms at the molecular level. The alternation of activity and rest over a period of 24 h (or rest-activity cycle) was observed in drosophila, fish and mammals including rodents (rats, mice, hamsters) and humans. In mammals, this circadian rhythm is generated by a master central clock, mainly reset by ambient light, and is located in the suprachiasmatic nuclei (SCN) of the hypothalamus [33,34]. Circadian regulation of all biological functions is processed through direct or indirect signals (cyclic hormone production) between the suprachiasmatic nuclei and different body structures (brain regions, organs). The body has peripheral clocks located in each organ (heart, lung, liver, muscles, kidneys, retina, etc.) that optimize the function of each organ according to the environmental context allowing therefore adaptation of the organism to environmental changes. Thus, the circadian clocks network has an adaptive function [35]. Both central and peripheral clocks are detectable though both cyclic gene expression and rhythmic physiological processes. These peripheral clocks work independently but must be re-synchronized continuously through the brain’s master clock acting as a real conductor. Furthermore, the neurohormone melatonin is involved in the synchronization (i.e., adjustment of the timing of existing oscillations) of peripheral oscillators; the nocturnal synthesis and release of melatonin by the pineal gland are controlled by the SCN master clock and inhibited by light exposure [36].

### 1.2. Clock Genes

The first gene known as being responsible for the circadian rhythm was found in drosophila; it is the *period* (*Per*) gene [37]. Subsequent research has highlighted another gene involved in the circadian rhythm: the *timeless* (*Tim*) gene [38,39,40,41].

Chemical mutagenesis performed in mammals such as mice allowed identification of the first clock genes: the *Clock* gene [42] (or *Npas2* in neuronal tissue), whose mutation was responsible for a lengthened rest–activity cycle. Other clock genes were then identified in the circadian regulation of the mouse: the *brain and muscle ARNT-like protein 1* (*Bmal1*) gene as a *Clock* partner, *Per1* and *Per2* genes, and *cryptochrome-1* (*Cry1*) and *Cry2* genes (genes encoding proteins involved in blue light reception in non-mammalian species).

The role of melatonin on the circadian oscillatory rhythms, and in particular as a neuroendocrine synchronizer of molecular oscillatory systems has been documented [36,43,44,45,46]. Studies in animal models showed that melatonin is involved in the regulation of circadian expression of several clock genes, such as *Per1*, *Per2*, *Bmal1*, *reverse erythroblastosis virus* (*REV-ERBα*), *Clock* and *Cry1*, in both central and peripheral melatonin target tissues. For example, in rat and mouse models, it has been shown that melatonin induces rhythmic expression of *Per1*, *Bmal1*, *Clock* and *Cry1* in the pituitary (*pars tuberalis*) [47,48]. It is noteworthy that *Per1* expression is undetectable in melatonin-deficient mice [49,50] and pinealectomy abolishes rhythmic expression of *Per1* in the *Pars tuberalis* (PT) and desynchronizes *Per1* and *Per2* expression in the SCN [51,52]. Moreover, melatonin synchronizes circadian oscillations in the cardiovascular system by influencing circadian rhythmic expression of both *Per1* and *Bmal1* in the rat heart [53]. In the pituitary of sheep, melatonin stimulates *Cry1* expression and suppresses other clock genes expression [54]. These effects are probably mediated by melatonin (MT) receptors, such as the MT1 receptors. Indeed, in the *pars tuberalis* of MT1 knockout mice, expression of *Per1*, *Bmal1*, *Clock* and *Cry1* was dramatically reduced but not changed in MT2 knockout mice [54]. Furthermore, in the hypothalamic SCN of rats, melatonin treatment induces phase advance of the nuclear receptor *REV-ERBα* expression [55]. In adipose tissue, melatonin synchronizes metabolic and hormonal function [56] by regulating *Per2*, *Clock* and *REV-ERBα* [57] (*REV-ERBα* is required for the daily balance of carbohydrate and lipid metabolism [58]). Finally, melatonin regulates oscillation of *Clock* genes in healthy and cancerous human breast epithelial cells [59] and induces a shift in the 24-h oscillatory expression of *Per2* and *Bmal1* in cultured fetal adrenal gland [60]. Taken together, these studies underline the major role of melatonin in the regulation of clock genes expression allowing the synchronization of central and peripheral oscillators.

In humans, homologues of *Clock*, *Bmal1*, *Per* and *Cry* were identified. The *Clock* gene, the only cloned circadian rhythm gene, is located on chromosome 4 [61]. There are other clock genes involved in circadian regulation, such as *retinoic acid receptor-related orphan receptor* (*ROR*)*-A* and *ROR-B*, *REV-ERB*, and *casein kinase-1* (*Ck1*)ε and *Ck1δ*, which control transcription of *Bmal1* gene. Additionally, the *albumin-D-site-binding protein* (*Dbp*) is also a circadian clock-controlled gene, involved in the circadian transcriptional regulation of several metabolic enzymes and some transcription factors. Some studies [25,26,27] report altered expression of certain clock genes in cases of shift workers (*Per1* and *Per2*), and free-running/constant conditions (*Per1*). However, most of the clock genes are affected in case of sleep-deprivation studies [62,63], highlighting the interaction between clock genes and sleep reported by Franken et al. [64,65]. Indeed, clock genes contribute to the homeostatic aspect of sleep regulation and mutations in some clock genes modify the markers of sleep homeostasis and an increase in homeostatic sleep drive alters clock genes expression in the forebrain [64]. It is noteworthy that the expression of clock genes is ubiquitous in humans (central nervous system, spleen, thymus, intestine, heart, lung, etc.), but also in animals (clock genes are found in most cell types among vertebrates).

### 1.3. Molecular Working of the Cellular Circadian Clock

The discoveries of the different clock genes led to the proposal of a molecular clock model based on a feedback loop running over 24 h [66]. The molecular mechanisms underlying the circadian rhythms are rather similar regardless of the species. They include enhancer elements, repressor elements and control loops involving phosphorylation-dephosphorylation, methylation, acetylation reactions and the specific protein dimerization [67,68]. In mammals, this molecular circadian system is present in the hypothalamic central clock, the suprachiasmatic nuclei, and in secondary clocks within the brain and peripheral organs. Light perceived by the retina leads to changes in transcription of certain genes of the circadian rhythm in the master clock and shows the ability of the body to adjust to a change in the cycle of the photoperiodic environment. This master clock synchronizes multiple peripheral circadian oscillators via mechanisms that remain to be better ascertained.

**Figure 1:**
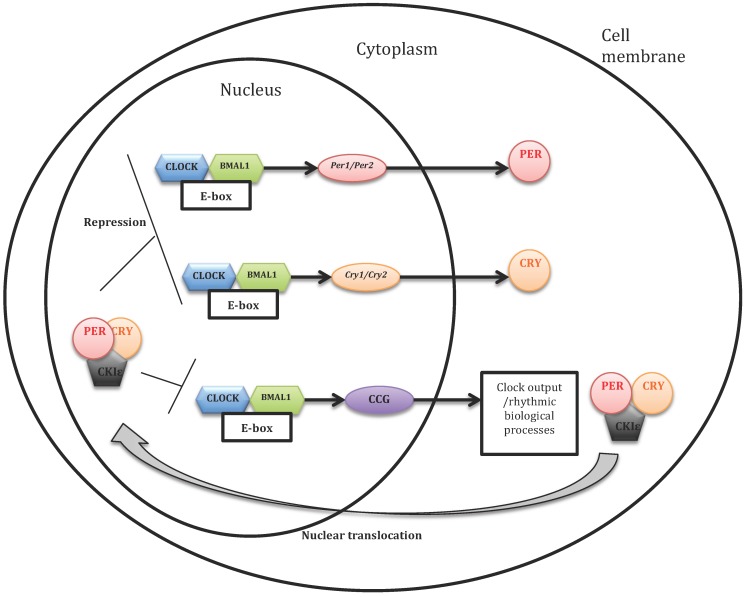
Model of the mammalian cell-autonomous oscillator (based on Lowrey and Takahashi, 2011) [69]. The transcriptional activators *circadian locomotor output cycles kaput* (CLOCK) and *brain and muscle ARNT-like protein 1* (BMAL1) stimulate the expression of *cryptochrome-1* (*Cry*) and *period* (*Per*) genes. The protein products of these genes are associated in the cytoplasm to form dimers that go into the core. There, they serve two functions: first, the repression of their own transcription, via the inhibition of CLOCK–BMAL1; and second, the activation of *Bmal1* gene, by a mechanism that remains to be discovered. These proteins are thus two regulating loops, one negative and the other positive. CLOCK and BMAL1 activate also the so-called clock-controlled genes (CCG) whose products transmit the rhythm information to the rest of the body via the output channels of the clock. Some proteins modulate the progression of control loops. Thus, casein kinase Iε (CKIε) can phosphorylate PER proteins, which destabilizes them and prevents their translocation into the nucleus.

The molecular loop of the circadian clock seems to imply in a general way two types of mechanisms: transcriptional (transcriptional regulation of genes at the DNA level, i.e., their copy in the form of RNA messenger) and post-transcriptional (regulating steps downstream of the transcription) (see Figure 1 [69]).

Transcriptional mechanisms: these mechanisms can be summarized by autoregulatory feedback loops. The first is a primary negative feedback loop: they rely on a pair of positive elements and a pair of negative elements. In mammals, the positive elements are two proteins, CLOCK and BMAL1 transcription factors which heterodimerize each other. The two negative elements are also two proteins, PERIOD (PER) and CRYPTOCHROME (CRY). In a neuron of the suprachiasmatic nucleus, there is little PER and CRY in the morning. The CLOCK–BMAL1 complex activates maximally the transcription of *Per* and *Cry* genes. However, the PER and CRY proteins do not accumulate immediately due to their instability, which allows the cycle not to be interrupted prematurely. Following interactions with other proteins they are gradually stabilized during the day, then they heterodimerize and finally migrate into the nucleus [70], where they inhibit the transcriptional activity of the CLOCK–BMAL1 heterodimer and thus their own transcription [71,72]. The *Per* and *Cry* genes being less and less active, the PER and CRY proteins are less and less produced. Their amount reaches a maximum at the beginning of night, and then decreases. At the same time, the CLOCK–BMAL1 heterodimer gradually regains its activity during the night. Thus, the molecular mechanism of the clock consists principally in a feedback loop comprising a positive component (with the CLOCK–BMAL1 heterodimer) and a negative component (with the PER–CRY heterodimer). A complete cycle of this loop lasts about 24 h. CLOCK and BMAL1 heterodimers are also involved in a daily transcription of many clock-controlled genes (CCGs) in different peripheral tissues [73,74,75]. In addition to the primary feedback loop, another regulatory feedback loop is formed by the orphan nuclear receptors REV-ERBα and RORα. In the nucleus, REV-ERBα competes with RORα for binding to the ROR-responsive element (RORE) in the *Bmal1* promoter. Whereas RORα activates transcription of *Bmal1*, REV-ERBα represses it. Consequently, the cyclic expression of *Bmal1* is achieved by both positive and negative regulation of RORs and REV-ERBs, respectively. This secondary feedback loop is called the “stabilizing loop”.

Post-transcriptional mechanisms: the proper functioning of the circadian loop requires that PER and CRY proteins disappear in due course, once they have served their purpose. As long as they inhibit the activity of positive elements (CLOCK and BMAL1), a new cycle cannot start. Several post-transcriptional processes thus affect the ability of PER and CRY to act on CLOCK and BMAL1. The most studied aspect is the modification of these proteins by phosphorylation and dephosphorylation. Several kinase proteins include PER and/or CRY among their targets, and each one of the two proteins can be phosphorylated on many distinct sites. They accelerate or slow down their transfer to the proteasome. Proteins to be degraded are “marked” by ubiquitin. Post-transcriptional regulations of the clock proteins of the main molecular loop make it possible to ensure intracellular traffic, functionality and degradation of clock proteins which are crucial for the functioning of the molecular loop over 24 h [76].

## 2. Relationships between Circadian Rhythms and Psychiatric Disorders

Several hypotheses can be raised regarding the relationships between the development of psychiatric disorders and problems of circadian rhythms, including altered sleep–wake rhythms:

(1) First, sleep problems may lead to cognitive impairments due to the effects of sleep deprivation and fatigue on learning and attention capacities, long-term memory, language development and emotions [77,78,79,80,81,82];
(2) Recent studies in cognitive and developmental psychology have highlighted the importance of rhythmicity and synchrony of motor, emotional, and inter-personal rhythms in early development of social communication; the synchronization of rhythms allows tuning and adaptation to the external environment [83]. Impaired circadian rhythms with an absence of synchronization of the circadian clocks network might alter the functioning of motor, emotional and interpersonal rhythms, leading to social communication impairments and vulnerability to psychiatric disorders with social communication deficit such as ASD or schizophrenia (for a review of literature on the importance of rhythmicity and synchrony of motor, emotional, and inter-personal rhythms in early development of social communication, see [83,84]). In addition, circadian rhythms involve sequences of continuities/discontinuities that might be important for typical fetal and child development in order to provide a secure environment (through stable and predictable regularities) but also variations allowing the individual to adapt to changes. Impaired circadian rhythms with no, little or irregular variability might lead to anxiety and difficulties in adapting to changes associated with restricted and repetitive interests observed in some psychiatric disorders described in this article such as ASD, schizophrenia and anxiety disorder;
(3) Clock genes control critical periods of brain development [85] and therefore, abnormal expression of clock genes might participate to neurodevelopmental disorders such as psychiatric disorders. It is noteworthy that only a few days of circadian rhythm impairments may impact the maturation and specialization of some brain structures at specific developmental periods; these abnormalities can alter the temporal organization of brain maturation and development [86];
(4) Circadian rhythm impairments may alter transcriptional and splicing regulation of Parvalbumin (PV) neurons, knowing that PV knockout mice (PV−/−) or heterozygous (PV+/−) mice showed autism behavioral phenotype [87]. More generally, circadian rhythm impairments may affect gene expression involved in synapse formation and brain maturation;
(5) Also, clock-controlled genes (CCGs) may have pleiotropic effects outside the molecular clock and have therefore more widespread impact on cognition, mood, and reward-related behaviors [88];
(6) Finally, circadian rhythm impairments (provoked or not by sleep problems) may also alter the adaptation of the individual to his/her environment and therefore his/her state of homeostasis. In this perspective, psychiatric disorders might reflect a loss of synchronization between the external environment’s rhythms and the individual’s internal rhythms, leading to major problems of adaptation for the individual and the appearance of psychiatric disorders. Single-nucleotide polymorphisms (SNPs) in core circadian clock genes have been associated with autism spectrum disorder [89], attention deficit hyperactivity disorder [90,91], anxiety disorder [92], major depressive disorder [93,94,95], bipolar disorder [95,96,97] and schizophrenia [98,99,100,101]. However, the causal relationship for these associations remains to be better ascertained. Circadian clock genes may affect specific aspects of psychiatric disorders through circadian control or through distinct regulation of downstream effectors.

The suprachiasmatic nuclei (SCN) coordinate the rhythms of other brain regions and peripheral organs like a pacemaker [102]. The sleep–wake cycle is the most documented example of the activity of the SCN, but endocrine, metabolic and immunological activities are also conducted by the SCN. For example, the glucocorticoids are regulated by the circadian cycle and affect the circadian rhythms of the amygdala [103,104]. Also, they regulate directly the clock genes [105,106].

We will explore the current state of research on this subject through five psychiatric disorders: bipolar disorder, anxiety and depression, attention deficit disorder, schizophrenia, and autism spectrum disorders.

### 2.1. Bipolar Spectrum Disorder

The mechanisms underlying the association between alterations in circadian rhythms and mood disorders are still unclear. However, current scientific studies provide food for thought. According to Jackson et al. [107], sleep disorder is the most common prodrome of mania and one of the six most common prodromes of depression in bipolar disorder (bipolar type I disorder is defined as an isolated manic episode, and bipolar type II disorder is defined as manic and depressive episodes) [108]. Between mood episodes, the sleep–wake cycle remains disrupted in patients with bipolar disorder who have some difficulty falling asleep and frequent night awakenings [109]. These patients have a significantly more eventide sleep pattern than control subjects, in other words, they go to bed later and have more difficulty getting up early compared to healthy controls [110].

McClung [111] studied the involvement of the *Clock* gene in manic episodes in mice. She compared *Clock* mutant mice to mice with a normal *Clock* gene. Mice in which the gene had been modified had mania-like behavior. Those mice were hyperactive, less anxious and less depressed. They slept less and showed greater brain activity in response to sugar water, cocaine and a mild electrical stimulation of the brain. When researchers added lithium in their water, these mice did not show their manic behavior anymore and started to act like the mice with a normal *Clock* gene. A lithium salt treatment could affect circadian rhythms by modulating the expression of clock genes [112,113,114,115]. Indeed, lithium salts might inhibit the expression of *glycogen synthase kinase 3β (GSK-3β)* gene [116], a gene involved in the brain’s biological clock [117]. Kaladchibachi demonstrated that both genetic and pharmacological reduction of *GSK-3* activity in mice have a specific effect on the circadian transcriptional oscillation consisting of *mPer2* period lengthening, indicating a delay in phase [116]. Moreover, it has been shown that *GSK-3β* is rhythmically expressed in the SCN and liver of mice, and that it undergoes a daily cycle in phosphorylation in vivo. Lithium chloride treatment inhibits the *GSK-3β* expression and results in a phase delay of *Clock* gene expression in fibroblasts [118]. Bipolar spectrum disorder has been associated with variations in the *Clock* gene. A single-nucleotide polymorphism (SNP) in the 3-flanking region of the *Clock* gene (3111 T to C) is associated with a higher recurrence rate of bipolar episodes [16]. This SNP was also associated in bipolar disorder and/or antidepressant treatment with sleep problems (insomnia and decreased need for sleep) [17,119]. Some studies reported other clock genes associated with bipolar disorder (such as *Bmal1* and *Per3)* [96]. Furthermore, a SNP in *Bmal1* and a SNP in *Tim* have also been identified as having a link with bipolar disorder [99]. One South Indian study showed that the occurrence of the five repeat alleles of *Per3* may be a risk factor for bipolar disorder onset in this ethnic group [120]. The coding region of *Per3* gene contains a variable number tandem-repeat (VNTR) polymorphisms which has been associated with diurnal preference, sleep structure and sleep homeostasis in healthy individuals. In a homogeneous sample of patients with bipolar type I disorder (occurrence of one or more manic episodes or mixed episodes), they observed that *Per3* VNTR influenced age of onset: earlier onset in homozygote carriers of *Per35* variant, later onset in homozygotes for *Per34*, and intermediate onset in heterozygotes. Sjöholm et al. [121] studied four single-nucleotide polymorphisms (SNPs) of *Cry2* gene in a cohort of bipolar patients, some with fast cycles. They observed that *Cry2* is associated with rapid cycling in bipolar disorder patients. Rapid cycling in bipolar disorder is defined according to the 5th version of Diagnostic and Statistical Manual of Mental Disorders (DSM-5) as four or more mood episodes in any combination or order within any year in the course of the illness. The A allele of rs10838524 (*Cry2* SNP) was significantly overrepresented among bipolar disorder cases with rapid cycling compared to controls. There was a significant trend in the association between the A allele of rs10838524 and rapid cycling (*p* = 0.0076) and this allele increased the risk for rapid cycling both in a homozygote and a heterozygote form (that is dominant model) compared to controls. Two closely related clock genes, retinoid-related orphan receptors α (*RORA*) and β (*RORB*) are involved in a number of pathways including neurogenesis, stress response, and modulation of circadian rhythms. A study reports that four intronic *RORB* SNPs showed positive associations with the pediatric bipolar phenotype and suggests that clock genes in general and *RORB* in particular may be important candidates for further investigation in the search for the molecular basis of bipolar disorder [122]. Further studies are necessary to better assess the role of *RORB* in bipolar spectrum disorder and explore if this gene is particularly relevant for this disorder compared to other clock genes.

Several genetic studies suggest that certain polymorphisms of clock genes are more common in bipolar disorder than in the general population [123,124]. The clock genes might therefore be viewed as genetic vulnerability factors to bipolar disorder [125]. These results need to be examined further.

The litterature review on clock genes and bipolar spectrum disorder is presented in Table 1.

### 2.2. Anxiety and Depression-Related Disorders

#### 2.2.1. Anxiety Disorder

A team of French researchers has evaluated a range of behaviors associated with psychiatric illnesses in mice in which two genes of the circadian clock, *Cry1* and *Cry2*, were investigated [126]. Their work highlighted the causal relationship between the disruption of encoding genes for cryptochrome proteins 1 and 2 (*Cry1* and *Cry2*) and behaviors associated with anxiety. Mice deficient for CRY 1 and 2 proteins show behavioral alterations characterized among other things by an abnormally high level of anxiety. These results indicate clearly that in addition to their critical roles in regulating the molecular clock; these proteins are directly involved in the control of emotional states.

#### 2.2.2. Major Depressive Disorder (MDD)

Sleep problems, in particular insomnia or hyperinsomnia, are parts of depression criteria and have been hypothesized to be under genetic control. Major depressive disorder, as well as seasonal affective disorder (winter depression and summer depression) and bipolar type I disorder provide excellent models for studying the molecular mechanisms of mood disorder. An important study published in 2013 first demonstrated dysfunctioning clock genes in brains from depressed humans compared to healthy controls [127]. Involvement of the clock genes in depression is also evident from several genetic studies. It has been reported that polymorphisms of clock genes appear in depressed patients [93,128,129,130,131,132].

Several SNPs in the *Clock* gene (T3111C, 3117 G to T, 3125 A to G) has been reported [127,128,133] to be associated with major depression and sleep disturbances (but some studies did not found any association between T3111C and sleep problems; [134,135]). More precisely, the two rare SNPs (3117 G to T and 3125 A to G) were associated with alternating phases of good sleep and insomnia over the course of a few days [133].

A Swedish team, Lavebratt et al. [94] reported in healthy controls a marked diurnal variation in *Cry2* mRNA levels; total sleep deprivation induced a 2.0-fold increase in *Cry2* mRNA levels. In patients with depressive state of bipolar disorder, sleep deprivation induced significantly decreased *Cry2* mRNA expression compared with healthy controls. In addition, *Cry2* mRNA levels were found to be lowered in blood mononuclear cells from depressed patients with bipolar disorder after total sleep deprivation in comparison to healthy controls, and *Cry2* gene variation was associated with winter depression in both Swedish and Finnish patients [94]. Deletion of *Cry2* gene lengthened the circadian period by approximately 48 min. This study [94] suggests that a *Cry2* locus is associated with vulnerability for depression, and that mechanisms of action involve dysregulation of *Cry2* expression. Associations between the gene *Cry2* and winter depression, but also dysthymia and bipolar type I disorder as seen previously, support the view that the *Cry2* gene has a role in mood disorders.

Shi et al. [132] found genetic polymorphisms in circadian genes, especially *Clock* and *Per3*, in major depressive disorder individuals. Authors propose that the impact of the *Clock* and *Per3* SNPs on transcription and/or expression may not be on the core circadian oscillator in humans, but on global output transcriptional pathways, that are mediated sex-dependently by the circadian system.

The litterature review on clock genes and depression related disorders is presented in Table 2.

#### 2.2.3. Familial Advanced Sleep Phase Syndrome (FASPS)

A good example of an abnormal circadian system in humans is the familial advanced sleep phase syndrome (FASPS), described in three families by Jones et al. [136]. FASPS is often associated with depression and anxiety. Affected individuals have a lead of a few hours of their sleep patterns and rhythms of temperature and melatonin, and a shorter period than healthy individuals in constant conditions. The joint efforts of several laboratories of the University of Utah in Salt Lake City have recently led to the description of *Per2* mutation in one of those families affected by FASPS. The mutation caused a substitution of serine by glycine in the *Per2* gene at position 662 [137]; this serine that can normally be phosphorylated by casein kinase Iε (CKIε). Inhibiting the action of CKIε accelerates the clock, which is indeed what is observed in FASPS patients. FASPS are also to be caused by a T44A missense mutation in human CK1δ, which causes hypophosphorylation of *Per2* [138,139]. Transgenic mice over-expressing the *hPer2* mutation exhibit shorter free-running periods, mimicking FASPS [140], and their phenotype is sensitive to CK1δ: increased dosage of CK1δ shortens their period further.

Recently, a team has identified a new mutation in the *hCry2* gene associated with FASPS. The mutation leads to replacement of an alanine residue at position 260 with a threonine. In mice, the *Cry2* mutation causes a shortened circadian period and reduced phase-shift to early–night light pulse associated with phase-advanced behavioral rhythms in the light-dark cycle [141].

#### 2.2.4. Seasonal Affective Disorder (SAD) and Delayed Sleep Phase Syndrome (DSPS)

Environmental changes like the seasons can be associated with depressive episodes in the inability of the circadian clock to adjust appropriately. Seasonal affective disorder (SAD) is defined as recurrent depressive disorder characterized by a seasonal pattern with, generally, an appearance in the fall or winter, apart from triggering external factor, and spontaneous disappearance in the spring or summer, even in the absence of treatment. SAD affects about 5% of the general population [142]. Lewy evokes the idea of a “phase delay of the endogenous circadian oscillator in relation to sleep-wake rhythm” [143], the equivalent of a delayed sleep phase syndrome (DSPS), as the cause of SAD. Genetic variants in *Npas2*, *Per2*, and *Bmal1* have been found to combine with the development of SAD [93,144].

Iwase et al. [145] reported that the SNP T3111C in the *Clock* gene was associated with morning or evening preference for activity and its frequency was decreased in DSPS. Futures studies are required to investigate the possible contribution of T3111C to DSPS susceptibility.

### 2.3. Attention Deficit Hyperactivity Disorder (ADHD)

Many sleep disorders are associated with attention deficit hyperactivity disorder (ADHD), both in adults and children. In particular, the following sleep problems occurring at sleep–wake transition were observed: bed-time refusal, delayed sleep-onset, and early awakenings [148]. Differences in sleep problems were found as a function of ADHD subtype: children with ADHD inattentive type (ADHD-I) had the fewest sleep problems and did not differ from controls, children with ADHD combined type (ADHD-C) had more sleep problems than controls and children with ADHD-I. Daytime sleepiness was greatest in ADHD-I and was associated with sleeping more (not less) than normal [149].

Several studies reported genetic associations between polymorphism (rs1801260) at the 3′-untranslated region (3′-UTR) of the *Clock* gene and psychiatric disorders [17,90] (see Table 3). Concerning the association between the rs1801260 polymorphism and ADHD, Kissling et al. [90] found at least one T-mutation being the risk allele in Caucasians of western European origin and German background.

Xu et al. [91] conducted a study which investigated a previously reported discovery of an association between a single nucleotide polymorphism in the 3′-UTR region of the *Clock* gene (rs1801260) in two independent samples of ADHD probands from UK and Taiwan (aged 5 to 15). They found a significant over-transmission of the T allele of rs1801260 SNP, which is associated as a risk allele for delayed sleep phase syndrome [145] in ADHD cases in the Taiwanese population. No association was observed between this polymorphism and ADHD in the UK sample. However, they did find evidence for increased transmission of the T allele of rs1801260 in the Taiwanese samples. Their findings support the hypothesis that genetic variation in the 3′-UTR region of *Clock* gene might be a risk factor for the development of ADHD, particularly in the Taiwanese sample studied. Therefore, more functional polymorphisms of this region should be investigated in other independent studies using larger samples.

### 2.4. Schizophrenia

Schizophrenia is often associated with sleep alterations [88,150], observed also in untreated patients [151]. Patients with schizophrenia have a desynchronization between the sleep–wake rhythms and melatonin profiles [88,152,153], the rhythms of body temperature [154], and serum levels of tryptophan and prolactin [155]. These anomalies suggest strongly disturbed circadian rhythms in these patients [156]. Studies of polymorphisms of clock genes or alterations in the regulation of these genes in schizophrenia are rare. The litterature review on clock genes and schizophrenia is presented in Table 4.

The expression of *Per1* mRNA in the temporal lobe in individuals with schizophrenia decreases significantly compared with age-matched healthy controls [98]. Another study suggested that *Per3* but not *Per2* abnormalities were associated with schizophrenia [99]. In a sample of 145 Japanese individuals with schizophrenia compared with healthy controls, it was reported that the T3111C polymorphism of the *Clock* gene presented a transmission bias. The T3111C polymorphism of the *Clock* gene might be associated with aberrant dopaminergic transmission in the suprachiasmatic nucleus, which is presumably involved in the pathophysiology of schizophrenia [157]. More recently, two studies focused on white blood cells and fibroblast from smaller samples: Sun et al. [100] reported altered expressions of *Per1*, *Per2*, *Per3* and *Npas2* in white blood cells in individuals with schizophrenia. Indeed, compared with healthy controls, schizophrenia patients presented disruptions in diurnal rhythms of the expression of *Per1*, *Per3*, and *Npas2*, accompanied by a delayed phase in the expression of *Per2* and by a decreasing in *Per3* and *Npas2* expression. Johansson et al. [101] reported a loss of rhythmic expression of *Cry1* and *Per2* in fibroblasts from individuals with schizophrenia compared to cells from healthy controls.

To date, the associations between clock genes and schizophrenia are not clear. 

### 2.5. Autism Spectrum Disorder (ASD)

Surveys of parents show that the prevalence of sleep problems in ASD is 50%–80% compared to 9%–50% in age-matched typically developing children [7,8,9,10,11,12,13,14]. Recent results showing abnormal melatonin secretion in ASD children may change the initial disregard of these disorders and suggest a possible key role of the clock and circadian regulations in ASD. Several independent groups detected abnormal melatonin levels in ASD [158,159,160,161,162,163]. These studies conducted on independent autism samples and using different methodologies indicate that abnormally low melatonin level is a frequent trait in ASD. More precisely decreased nocturnal as well as diurnal levels have been reported in individuals with ASD; trial studies support therapeutic benefits of melatonin use in ASD [83,84,161]. Nevertheless, both the underlying cause of this anomaly and its relationship with ASD (cause or consequence?) remain still unexplained.

Many studies have advocated a genetic etiology for autism [164] involving in particular synaptic genes related to synaptic cell adhesion molecules NLGN3, NLGN4, and NRXN1 and a postsynaptic scaffolding protein SHANK3. This protein complex is crucial for the maintenance of functional synapses as well as the adequate balance between neuronal excitation and inhibition. Among the factors that could modulate this pathway there are genes controlling circadian rhythms. However, no direct association has been reported between clock genes and NLGN3, NLGN4, NRXN1 and SHANK3 in humans, but epistasis mechanisms cannot be ruled out. SHANK3 expression is modulated by melatonin concentration and the modulation is brain structure-dependent [165]. Concerning relationships between sleep disorders and NLGN3, NLGN4, NRXN1 and SHANK3 could be a pleiotropic effect on sleep of genes associated with brain disorganization. It is noteworthy that possible interplay of synaptic and clock genes may increase the risk of ASD [166]. The involvement of clock genes in ASD was first suggested by Wimpory et al. [167] who stated the hypothesis that anomalies in clock genes operating as timing genes in high frequency oscillator systems may underline timing deficits that could be important in the development of autism spectrum disorder, notably in autistic communication impairment. To test this hypothesis, Nicholas et al. [89] screened single-nucleotide polymorphisms in 11 clock/clock-related genes in 110 individuals with ASD and their parents. A significant allelic association was detected for *Per1* and *Npas2*. It should be noted that it was a small population and results were not significant after correction for multiple testing. However, the association between *clock* genes and ASD was confirmed by a more recent study [168] reporting also mutations in other circadian clock genes (*Per2*, *Per3*, *Clock*, *Bmal1*, *Tim*, *Cry1*, *Cry2*, *Dbp* and *Ck1ε*) in ASD patients. Taken together, these findings suggest that the circadian rhythm abnormalities observed in ASD may be linked to abnormalities of the circadian clock genes. Finally, it is noteworthy that clock genes are related to the protein association network described in ASD by Roubertoux and Tordjman [169] either through direct gene × gene interactions or protein × protein interactions.

The litterature review on clock genes and autism spectrum disorder is presented in Table 5.

## 3. Conclusions

Circadian clocks enable organisms to anticipate temporal organization of biological functions in relation to periodic changes of the environment, and to adapt consequently their behavior. The genes *Clock*, *Per*, *Cry* and *Bmal1* are currently the major clock genes identified in humans as being involved in the rhythmicity and timing of biological rhythms at the molecular level. Their alteration involves changes to the 24-h rhythm through poor synchronization between the endogenous circadian rhythms and the sleep-wake cycle, and act especially on sleep disorders. These are often early symptoms of altered sleep–wake rhythms at the onset of psychiatric disorders, especially for mood disorders. Furthermore, impairments in the four major clock genes (*Clock*, *Per*, *Cry* and *Bmal1*) were found for bipolar disorder, depression-related disorders, autism spectrum disorder, and impairments in some of these major clock genes were also reported for schizophrenia (*Clock*, *Per* and *Cry*), anxiety disorder (*Cry*) and attention deficit hyperactivity disorder (*Clock*). In addition, other *clock* genes were associated with these psychiatric disorders, such as *Npas2* (winter depression, autism spectrum disorder and schizophrenia), *RORA* and *RORB* (bipolar disorder) or *Tim*, *Dbp* and *Ck1ε* (autism spectrum disorder). The associations of identical clock genes with these different psychiatric disorders suggest that they may share similar pathways and etiopathogenic mechanisms. It highlights the interest and need to study these mental disorders through a transnosographic and multidimensional approach focusing on depression, anxiety and stress responses.

The cascading effects resulting from altered clock genes, poorly understood so far, could participate in sleep problems and the emergence of symptoms present in certain psychiatric disorders through, as discussed in the article, impaired regulation of circadian rhythms and emotional states with neurodevelopmental effects (including impaired control of the temporal organization of brain maturation, neurogenesis, synapses formation/functioning and brain specialization at specific developmental periods). Inversely, sleep problems can alter the expression of clock genes and contribute, as seen in this article, to the development of psychiatric disorders through cognitive effects of sleep deprivation and fatigue. More generally, alteration of clock genes (directly or indirectly through sleep problems) might lead to desynchronized and abnormal circadian rhythms (including sleep/wake rhythm but also other circadian rhythms such as neuroendocrine or body temperature rhythms) impairing in turn the synchronization between external and internal rhythms and therefore the adaptation of the individual to his/her internal and external environment with the development of psychiatric disorders. Future studies are required to better ascertain the underlying mechanisms of the relationships between clock genes, sleep disturbances and psychiatric disorders. Further research may open interesting perspectives with promising avenues for early detection and therapeutic intervention in psychiatric disorders.

## Figures and Tables

**Table 1:** Clock genes and bipolar spectrum disorder. RORα: retinoid-related orphan receptors α; RORβ: retinoid-related orphan receptors β.

Studies	Measure	Individuals with Psychiatric Disorder and/or Organisms Models (*n*)	Controls (*n*)	Results
Kaladchibachi et al. [116]	Cyclical expression of clock genes (*Per2*)	Mouse embryonic fibroblasts (MEFs)	-	Genetic depletion of glycogen synthase kinase 3 (GSK3) activity results in a significant delay in the cycling period of *Per2.*
McGrath et al. [122]	Genotyping and analysis of 312 single-nucleotide polymorphisms (SNPs) in *RORA* and 43 SNPs in *RORB*	Bipolar disorder (BD) children (*n* = 305)	Healthy parents (*n* = 306) Healthy individuals (*n* = 140)	Four intronic *RORB* SNPs showed positive associations with the pediatric bipolar phenotype.
Lavebratt et al. [94]	Assessment of *Cry2* gene expression before and after one night of sleep deprivation	BD individuals (*n* = 13)	Healthy individuals (*n* = 8)	*Cry2* mRNA levels are reduced and unresponsive to sleep deprivation in depressed patients with bipolar disorder.
Sjöholm et al. [121]	Analysis of four *Cry2* single-nucleotide polymorphisms	BD individuals in Sweden (*n* = 577); BD type I (*n* = 497); BD type II (*n* = 60); BD with rapid cycling (*n* = 155)	Healthy individuals (*n* = 1044)	Association between the circadian gene *Cry2* and rapid cycling in bipolar disorder.
Karthikeyan et al. [120]	Genotyping and analysis of *Per3* in blood samples	Bipolar type I disorder individuals in South India (*n* = 311)	Healthy individuals (*n* = 346)	The occurrence of the five repeat allele of *Per3* may be a risk factor for bipolar type I disorder onset in this ethnic group.

**Table 2:** Clock genes and depression related disorders.

Studies	Measure	Individuals with Psychiatric Disorder (*n*)	Controls (*n*)	Results
Takimoto et al. [146]	Daily variation of melatonin and cortisol, and daily expression of clock genes (*Per*, *Bmal1* and *Clock)* in whole blood cells	Individuals with circadian rhythm sleep disorder (*n* = 1)	Healthy male individuals (*n* = 12)	The peak phase of *Per1*, *Per2*, and *Per3* appeared in the early morning, whereas that of *Bmal1* and *Clock* appeared in the midnight hours in healthy male individuals.
Partonen et al. [93]	Analysis of sequence variations (single-nucleotide polymorphisms) in three core clock genes: *Per2*, *Bmal*, and *Npas2*	Depressed individuals (*n* = 189)	Healthy individuals (*n* = 189)	Variations in the three circadian clock genes *Per2*, *Bmal*, and *Npas2* are associated with winter depression.
Utge et al. [147]	Analysis of 113 single-nucleotide polymorphisms of 18 genes of the circadian system	Depressed individuals (*n* = 384)	Healthy individuals (*n* = 1270)	Significant association between *Tim* variants and depression with fatigue in females, and association to depression with early morning awakening in males.
Lavebratt et al. [94]	Genotyping of single nucleotide polymorphism of the *Cry2* gene	Depressed individuals with bipolar disorder (*n* = 204)	Healthy individuals (*n* = 2017)	The *Cry2* gene was significantly associated with winter depression in both samples.
Kovanen et al. [130]	Genotyping of 48 single-nucleotide polymorphisms in *Cry1* and *Cry2* gene	Individuals with dysthymia (*n* = 136)	Healthy individuals (*n* = 3871)	Four *Cry2* genetic variants (rs10838524, rs7121611, rs7945565, rs1401419) are significantly associated with dysthymia.
Hua et al. [131]	Genotyping of single nucleotide polymorphisms (SNPs) of *Cry1* rs2287161, *Cry2* rs10838524 and *Tef (thyrotroph embryonic factor)* rs738499	Chinese individuals with major depressive disorder (MDD) (*n* = 105)	Chinese healthy individuals (*n* = 485)	The polymorphisms of *Cry1* rs2287161and *Tef* rs738499 are associated to major depressive disorder.
Shi et al. [132]	Genotyping of 32 genetic variants from eight clock genes	Major depressive disorder individuals (*n* = 592)	Healthy individuals (*n* = 776)	Genetic polymorphisms in circadian genes, especially *Clock* and *Per3*, influence risk of developing depression in a sex- and stress-dependent manner.

**Table 3:** Clock genes and attention deficit hyperactivity disorder (ADHD).

Studies	Measure	Individuals with Psychiatric Disorder (*n*)	Controls (*n*)	Results
Kissling et al. [90]	Analysis of polymorphism (rs1801260) at the 3’-untranslated region of the *Clock* gene	ADHD individuals (*n* = 143)	Healthy individuals (*n* = 143)	Significant association (*p* < 0.001) between genotype and ADHD-scores of the adult ADHD assessments, and the rs1801260 polymorphism with at least one T-mutation is the risk allele.
Xu et al. [91]	Analysis of polymorphism (rs1801260) at the 3’-untranslated region of the *Clock* gene in ADHD using within-family transmission disequilibrium test	Two clinical ADHD samples: United Kingdom (UK) sample: (*n* = 180); Taiwan sample: (*n* = 212)	Both parents or mother alone or father alone UK sample: (*n* = 296); Taiwan sample: (*n* = 326)	Increased transmission of the T allele of the rs1801260 polymorphism in Tawainese samples.

**Table 4:** Clock genes and schizophrenia. REV-ERBα: reverse erythroblastosis virus; Dbp: albumin-D-site-binding protein.

Studies	Measure	Individuals with Psychiatric Disorder (*n*)	Controls (*n*)	Results
Takao et al. [157]	Analysis of 3111C single nucleotide polymorphism of the *Clock* gene	Individuals with schizophrenia (*n* = 145)	Healthy individuals (*n* = 128)	Individuals with schizophrenia had a significantly higher frequency of the C allele compared to controls.
Sun et al. [100]	Relative expression of clock gene mRNA: *Per1*, *Per2* and *Per3* in blood samples	Individuals with schizophrenia (*n* = 13)	Healthy controls (*n* = 15)	Individuals with schizophrenia presented disruptions in diurnal rhythms of the expression of *Per1*, *Per3*, and *Npas2 c*ompared with healthy controls, accompanied by a delayed phase in the expression of *Per2* and by a decrease in *Per3* and *Npas2* expression.
Johansson et al. [101]	Analysis of *Clock*, *Bmal1*, Per1, *Per2*, *Cry1*, *Cry2*, *REV-ERBα* and *Dbp* in fibroblasts from skin samples	Individuals with chronic schizophrenia under neuroleptic medication (*n* = 11)	Healthy individuals (*n* = 11)	Loss of rhythmic expression of *Cry1* and *Per2* in fibroblasts from individuals with schizophrenia compared to cells from healthy controls.

**Table 5:** Clock genes and autism spectrum disorder (ASD).

Studies	Measure	Individuals with Psychiatric Disorder (*n*)	Controls (*n*)	Results
Nicholas et al. [89]	Screening of eleven clock/clock-related genes	High-functioning ASD individuals (*n* = 110)	Healthy parents (*n* = 220)	Significant association for two single-nucleotide polymorphisms in *Per1* and in *Npas2*.
Yang et al. [168]	Direct sequencing analysis of the coding regions of 18 canonical clock genes and clock-controlled genes	ASD individuals with sleep disorders (*n* = 14); ASD individuals without sleep disorders (*n* = 14)	Healthy individuals (*n* = 23)	Mutations in circadian-relevant genes (specifically *Per1*, *Per2*, *Per3*, *Clock*, *Npas2*, *Bmal1*, *Tim*, *Cry1*, *Cry2*, *Dbp* and *Ck1ε*) affecting gene function are more frequent in individuals with ASD than in controls.
